# Application of multivariant decision tree technique in high performance football: The female and male corner kick

**DOI:** 10.1371/journal.pone.0212549

**Published:** 2019-03-11

**Authors:** Rubén Maneiro, Claudio A. Casal, Antonio Ardá, José Luís Losada

**Affiliations:** 1 Department of Science of Physical Activity and Sport, Pontifical University of Salamanca, Salamanca, Spain; 2 Department of Science of Physical Activity and Sport, Catholic University of Valencia, San Vicente Martir, Valencia, Spain; 3 Department of Physical and Sport Education, University of A Coruña, A Coruña, Spain; 4 Department of Social Psychology and Quantitative Psychology, University of Barcelona, Barcelona, Spain; University of L'Aquila, ITALY

## Abstract

The use of multidimensional statistical technique based on decision trees is of recent application in sports science. In the case of football, this technique has not yet been sufficiently proven. The aim of the present study was to search for different success models for the corners in the FIFA World Cup 2014 and FIFA Women's World Cup 2015. For this, the statistical analysis focused on the search for classification models for the different criteria considered (shot, shot between the three posts and goal), based on the creation of different decision trees that allow the most important variables to be identified quickly and efficiently. For this, 1117 corners were collected between the two competitions, performed in 116 international matches. It has been possible to establish multivariate models for the "shot" and "shot between the three posts" criteria, allowing, in some cases, to quadruple the potential for offensive success. On the other hand, we have been able to identify significant differences in the male and female model of execution. These findings suggest the need to continue deepening the study of tactical behavior in women's soccer from a multivariate perspective, and also propose a better optimization of the management and training of this type of actions for both male and female football. In addition, it has allowed to test the decision tree statistical technique in the analysis of high performance football, with satisfactory results and of great relevance in the applied field.

## Introduction

Human science is more about destroying errors than about discovering truths. This quotation from the Greek philosopher Socrates could well apply to football science. The human being has sometimes used interpretations rather than certainties to describe the phenomena’s reality that occur in sport. Football has been a faithful witness of this maxim, where certain conjectures, which are far from the scientific method’s strictness [1, 2], are presented with great arrogance. Only by invalidating erroneous paths, in favour of verified truths, will this lead to what we call truth.

It is necessary to validate or exclude approaches and constructs, diminishing the resignation of the apparently non-measurable game. Topics like "football is football" or "the state of form is not good" are increasingly distant thanks to the push of science and data. Thanks to researchers and their concern to know beyond the visible with the naked eye, today we can empirically affirm that in football there is no perfect game system [3]; that a decline in performance is not only explained by physical parameters [4], that ball possession is still in debate as a performance indicator [5, 6], and that not all players should train the same, but must attend to specific demarcations [7] and different motor skills [8, 9].

Football is composed of two distinct phases that give the game meaning and personality. The first is the dynamic phase [10], moderately studied in scientific literature and occurs when the ball is in play. The second is the static phase [11] or stationary action, where the game resumes after a regulatory break.

As for stationary actions, available results should still be taken with caution. The lack of consensus in scientific literature shows this. The few studies available to date have not yielded conclusive results, and on some occasions the methodological filter has not been effective. Carling et al. [12], Wallace et al. [13] or Yiannakos et al. [11] collect descriptive results, which, while providing valid and interesting results, hardly explain the complex reality of the actions studied. A common conclusion of these works relates these actions with a high goal flow, which does not correspond to results of comparative or explanatory work [14, 15]. On the other hand, regarding these actions in women's football, and despite the fact that scientific literature has been increasing in recent years [16–18] it is not possible to refer profound studies on this type of actions.

These actions are included in the rules and appear consistently in the game, in the form of fouls, penalties, serve, goal kicks and corner kicks, and represent a frequent casuistry during matches. In men's football, there are 110 such actions per game (one every 45 seconds), representing 97% of all interruptions, comprising 41% of the 90 minutes match [19].

These actions have certain advantages for the team in charge of executing them. Unlike the game’s dynamic part, the player in charge of resuming the game in the stationary ball actions is in a stable situation, with a high contextual certainty, being the owner of the game’s restart, and where rivals must respect a certain distance with the executor player, which gives him a temporary advantage, which in the game’s dynamic context does not exist [20]. Time advantage is very important in soccer, where time and space reduction is a proven defensive attribute [21].

The corner kick is one of the actions that is more consistently repeated. The work of Sainz de Baranda et al. [15] has concluded that 10 corner kicks are executed per game, and that only 1 out of 4 are shot, data also corroborated by the works of [22], [12] and [23]. Other studies [14] gather that only 2.3% finish in goal and despite this low efficiency, 76% of them have been transcendental in the final result, giving valuable points to the teams.

About the methods that teams use to execute these actions, there is a great lack of consensus among the scientific community. On the one hand, descriptive works such as Pulling, Robins & Rixon [24] and Schmicker [25] have found that direct sendings to the area in search of a quick completion are the best option, being the ratio between the number of corners and goals very low, with relative values close to 40:1. On the other hand, comparative or bivariate works [26], affirm that goal is barely achieved in 2.5% of these actions, finding a significant association between the goal and the coordinated dynamics between the attacking players. On the other hand, the studies Arda et al. [20] and Casal et al. [14], by means of different binary logistic regression analysis, have proposed execution models based on short sendings, with the intervention of 3–4 players and with constant space creation and occupation movements, as the best way to achieve a shot or a goal. In their work they have been able to demonstrate that, with this type of execution, goal probability would almost quadruple.

The low success percentage of these actions, in contrast to the high regularity with which corners are shown during football matches, indicates that we are facing actions of high complexity and entropy, where the high number of actions to coordinate between the offensive players, drastically reduces the percentage of shot and goal. Therefore, and in view of the data presented, a thorough study, and with an effective analysis technique, is justified.

In the present work, a multivariate interaction analysis based on the creation of decision trees was carried out, with the aim of proposing different multivariate success models for the corner kick executed in women's and men's soccer. The use of decision trees in sports science is novel and has hardly been put to the test in the scientific study of football [27]. It is an efficient classification technique, based on the information collection, it allows to reduce the uncertainty or entropy, allowing to identify quickly and efficiently the most important variables that act as predictors. In addition, it is particularly well suited to the study of football, since its nature as a complex dynamic system [28, 29] requires an effective tool, capable of selecting and simplifying the complex interactions between players.

## Method

### Design

Observational methodology was applied, as it is most suitable due to its main characteristics of habitual context, spontaneity, and perceptivity [30], which are totally feasible in the sports field, and specifically in professional football.

This work is located in quadrant IV, and the observational design to which it is adjusted is nomothetic (plurality of units), intersessional (multiple sessions over time) and multidimensional (simultaneous and concurrent consideration of several response levels, reflected in the observation instrument), according to Anguera et al. [31] and Sánchez-Algarra et al. [32]

Systematic observation has been non-participant and active, and an 'all occurrences' observational sampling has been used [33, 34].

### Participants

In this study the sampling unit has been the corner kicks in high level football competitions, specifically 1117 corners were analysed in the 116 matches of the FIFA World Cup 2014 (n = 64) and FIFA Women's World Cup 2015 (n = 52). Inclusion criteria can be found in Casal et al. [14]. The sample of corners selection has considered the location of the matches (all matches are played in neutral field), as were studying the best selection teams worldwide.

### Instruments

An observation instrument proposed by Casal et al. [14] was used, consisting of a combination of field formats and categories systems, where the dimensions and categories that make up the instrument can be consulted (Table 1).

**Table 1:** 

VARIABLES	
Time (T)	0–30’
	31–60´
	61´-90
Laterality of corner (LS)	Right (D)
	Let (L)
No. of attackers	2–3
	4–5
	6 or more
Interaction context (COI)	Inferiority (IN)
	Equality (IG)
No. of defenders on the posts	0
	1
	2
Delivery of ball (EDF)	Direct (D)
	Indirect (I)
Path of ball (TB)	Ground (G)
	Air (A)
Type of marking (TD)	Individual (I)
	Zone (Z)
	Combined (C)
No. of intervening attackers	1–2
	3–4
End zone of the corner kick	Neat post (NP)
	Far post (FP)
Offensive organization (MOO)	Static
	Dynamic
Match status (MS)	Winning (W)
	Drawing (D)
	Losing (L)

Software used for the analysis is The R program, with the package “Classification and Regression Trees”. R package version 1.0–37. Data was collected and coded through LINCE software [35].

### Procedure

Matches were recorded from TV emitted images and were registered and analyzed post-event. Because the video recordings were public, confidentiality was not an issue and authorization was not required from the players observed or their representatives.

Eight observational sessions were held for the observers training, following Losada & Manolov [34] criteria, applying the consensual agreement among observers criterion, so that recording only happened when agreement took place. The four selected observers had experience as soccer coaches, three of whom were PhDs in Sports Sciences and experts in observational methodology. Prior to the coding process, observers were trained for two weeks to become familiar with the observation instrument.

Data quality control was carried out by an interobserver agreement analysis using the Cohen's Kappa coefficient (1960) for each criterion, average value (or overall) being very good (.93) according to the Fleiss et al. [36] scale.

A multidimensional analysis based on decision trees was proposed, which is one of the most efficient supervised classification techniques. When dealing with non-parametric methods, it is not necessary to comply with any specific distribution, and after the creation of a tree, observations are grouped in the terminal nodes. To predict a new observation, the tree is traversed according to the value of its predictors until it reaches one of the terminal nodes. In the case of classification, the mode of the response variable is usually used as a prediction value, that is, the most frequent class of the node. In addition, it can be accompanied by the percentage of each class in the terminal node, which provides information on prediction confidence.

The decision trees technique appears in the 50s, within the information theory. The concepts and formal aspects were developed by Shannon [37]. Decision trees can be used to generate expert systems, which use a joint probability distribution of a group of variables, to describe dependency relationships between them, and draw conclusions using probability theory formulas (Markov and Bayesian networks). They constitute an instrument of analysis that allows in principle to express graphically [38], and subsequently, under a mathematical schematization, the different paths, variables, causes and effects susceptible of materializing as a result of the actions derived by the participating individuals, that under conditions of uncertainty and risk, elements of a stochastic or random type converge in each phase.

The model used in this work is an algorithm for the reduction of dimensionality, with the intention of discovering patterns in which data are organized and then formulating predictions based on probabilities. It is a predictive model that can be used to represent regression models and classifiers. This decision tree technique is formed by graphical representations in the form of diagrams arranged in logical temporal sequences, with a perfectly branched structure, through which an attempt is made to represent a modeled form, of all the possible options of an action, linked to their respective occurrence probabilities. This model facilitates decision making, especially when there are multiple chains of options.

The tree building process tends to reduce training error quickly, that is, the model adjusts very well to the observations used as training. Therefore, *overfitting* is generated that reduces its predictive capacity when applied to new data. The reason for this behavior lies in the ease with which trees branch out by acquiring complex structures. In fact, if the divisions are not limited, every tree ends up adjusting perfectly to the training observations creating a terminal node by observation. There are two strategies to prevent the problem of tree *overfitting*: limiting the size of the tree and the *pruning* process.

The final size that a tree acquires can be controlled by stop rules that stop the division of the nodes depending on whether certain conditions are met or not. The pruning process consists of generating large trees, without stopping conditions beyond those required by computational limitations, and then pruning them, maintaining only the robust structure that achieves low error and reduces the variance of the model.

In this study, different criteria were constructed: Criterion 1: "shot", Criterion 2: "shot between posts" and Criterion 3: "goal" in the FWWC2015 and FWC 2014 competitions with the objective of building decision trees, which depending on a variable set predict the values of the criteria.

In the creation of the decision trees, 20 variables were used without including the three criteria and the type of competition, with a total of 1035 observations between the two competitions. Once the observations were classified according to competition, 587 observations were obtained for the men's world cup and 448 for the women's world cup. Each of these groups is divided into two sets, one with 70% of observations (training set), and the second with the remaining 30% (test set) to apply the cross-validation technique and get a better model, avoiding *overfitting*.

With all three criteria and working with the training set all variables were taken and the models for the FWC2014 and FWWC2015 were raised using decision trees that were pruned to obtain the most optimal, without variables that do not provide information.

## Results

### Criterion 1 FIFA Male World Cup 2014

**Fig 1:**
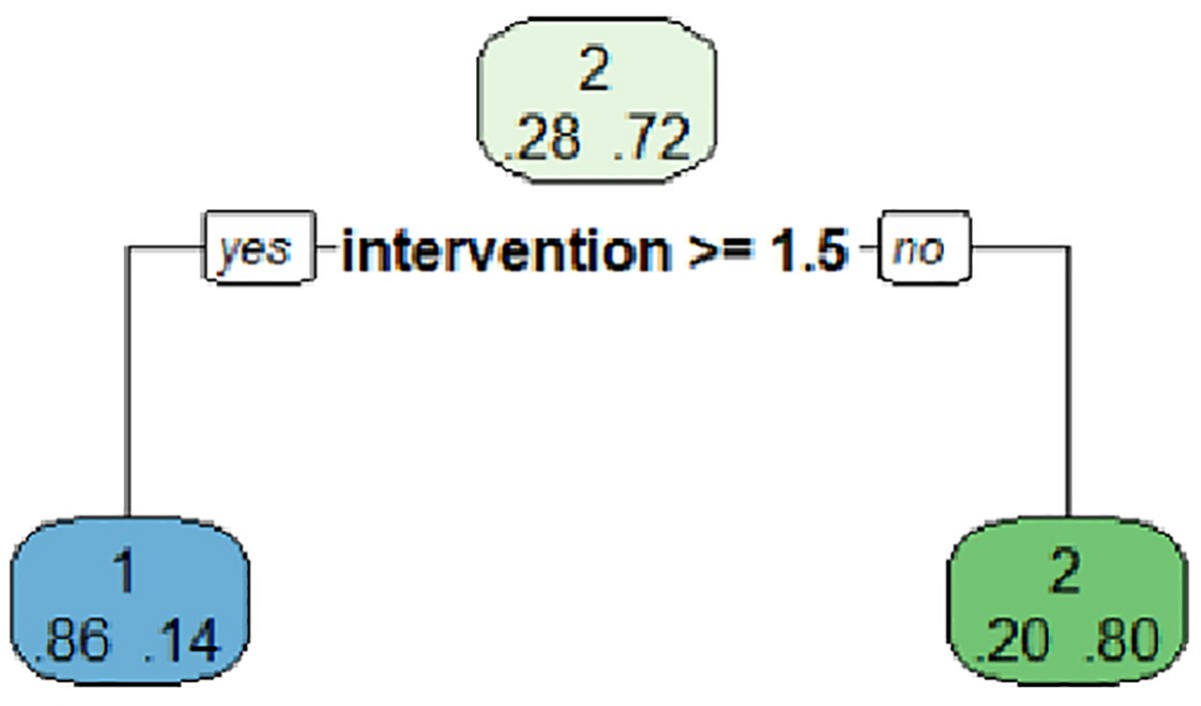
Criterion1 world male shows test.

Using the "test" set and once the model is optimized, the tree starts with the "shot" root node where it is observed that the category with the highest probability is the "no shot" (2) with a probability of 0.72 while the "shot" (1) has a probability of 0.28. The algorithm includes the variable "intervention" with the value 2 (> = 1.5) that corresponds to the category "3–4 players". In the case that this category doesn’t happen (reference "1–2 players") the probability of "shot" (2) is 0.2 and that of "no shot" is 0.8. If the category is given (3–4 players) the probability of "shot" (1) is 0.86, while the probability of "no shot" is 0.14. Both nodes are terminals, and the most interesting one is the "shot" branch (Fig 1).

This model can be evaluated in prediction terms (Table 2), so it is verified that it has predicted that there is "shot" a total of 55 times when there really have been, and 25 times when there really have not been. The model classifies the "no shot" in 135 times when really there is "shot", and 409 times that "no shot" when there really has not been a shot.

**Table 2:** 

Pred. \ Obs.	Shot	No shot
Shot	55	25
No shot	135	409

Finally, the effectiveness of the model is evaluated, adding all the successes and dividing by the number of predictions, obtaining a 74.36% effectiveness of successes.

### Criterion 1 FIFA Women's World Cup 2015

Following the first criterion "shot" and working with the "test" set taking all the variables a model is proposed for the women's world competition with an optimized decision tree.

The optimized decision tree is presented using the "test" set. It is observed that the probability of "no shot" is the most usual with a 0.73 while the "shot" is 0.27. The intervention variable is introduced with the category 3–4 players. In the case that this category is not given, the probability of “no shot” is 0.78 while the probability of “shot” is 0.22. If category presence is affirmative there is a 0.62 probability of “shot” versus 0.38 of “no shot”. Next variable introduced by the algorithm is "Time" in the category "0–30" and in this case the probability of "shot" is 0.81 while the probability of "no shot" is 0.19. This node would be terminal. For the categories "31–60" and "61–90" the probability of "shot" is 0.55 and "no shot" 0.45. Following this branch the algorithm includes the variable "No. Of Defenders On The Post" in the category "2 players"(> = 1,5) with a "shot" probability of 0.45 and a “no shot" probability of 0.55. And in the case of having the category "1 player" the "shot" probability is 0.62 and "no shot" probability of 0.38, configuring two terminal nodes. Finally, the variable "Zone To Which Pass Is Made" is included in its "far post" category, in this case, the probability of "shot" is 0.67 compared to 0.33 of "no shot". In the case that the "near post" category is not given, the probability of "shot" is 0.35 and "no shot" is 0.65. These last two nodes are terminals.

**Fig 2:**
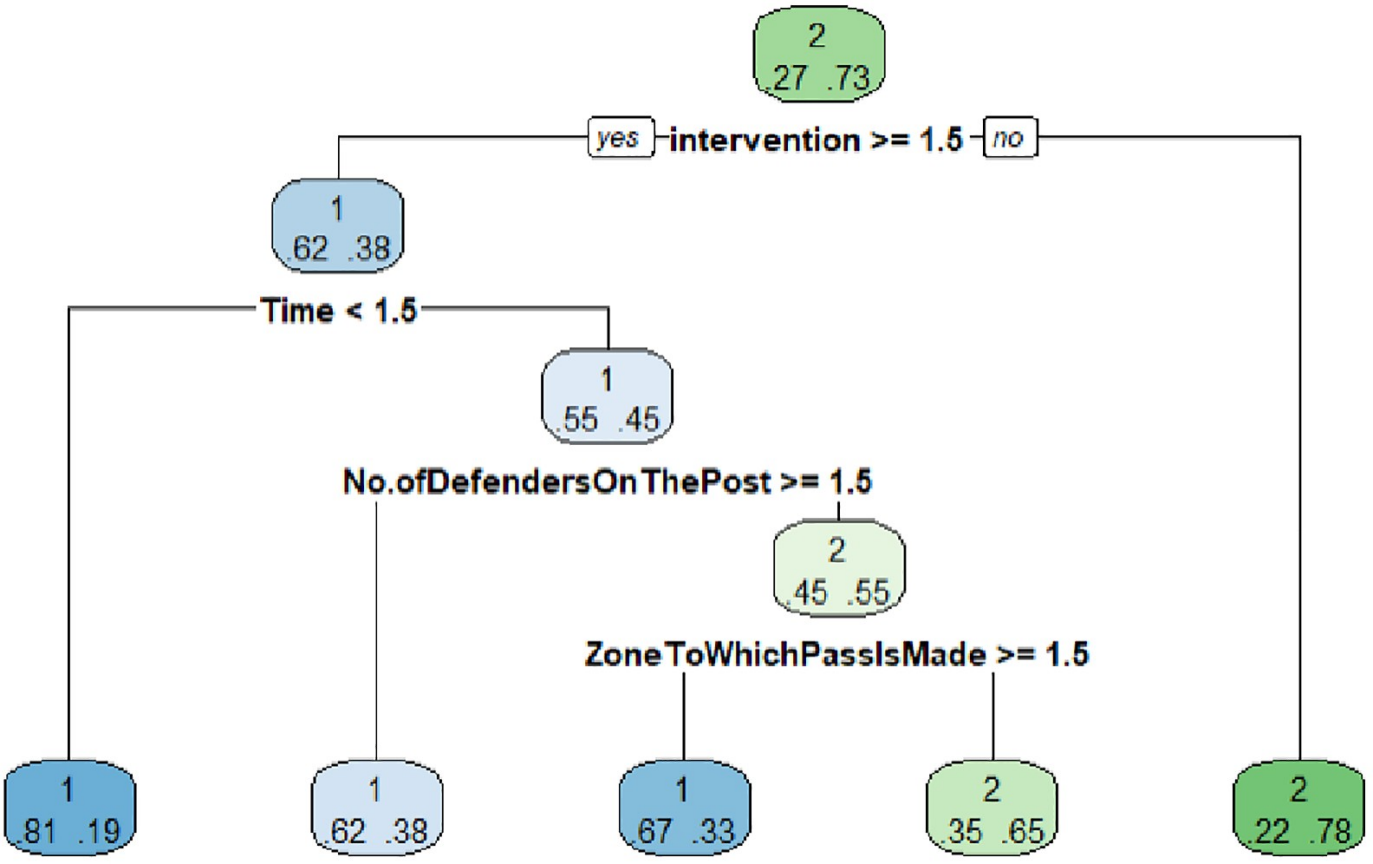
Criterion1 world female shows test.

From this tree we are interested in the branch whose terminal node is the realization of the "shot". Starting from a probability of 0.27, with an intervention of 3–4 players, combined with a "far post" shooting zone and a N°. of Defenders On The Post with 1 player, where the probability of "shot" is increased to 0.67 (Fig 2).

In prediction terms, the model predicts 50 “shots" and 50 "shots" have been observed. However, he predicts 22 "shots" when they have really been "no shot”. As for the "no shots", model predicts 140 when they have really been "shots" and predicts 412 "no shots" when they have really been 412 "no shots" (Table 3).

**Table 3:** 

Pred. \ Obs.	Shot	No Shot
Shot	50	22
No Shot	140	412

The effectiveness evaluation of the model, is of 74.03% successes.

### Criterion 2 FIFA Male World Cup 2014

Tree starts with the "shot between posts" root node, with a probability of 0.14, while the probability of not happening is 0.86.

The next variable included in the tree is the "intervention" with the category 3–4 players, in the case of not intervening 3–4 players, that is, intervention of 1–2 players the probability of "no shooting between posts" is 0.90, and the probability of "shooting between posts" is 0.10, terminal node. In the case of a 3–4 players (> = 1.5) intervention the probability of "shot between posts" is 0.38, while that of "no shot between posts" is 0.62.

Third node is formed by the variable "Shooting Area", which has two categories: "near post" (1), and "far post" (2). The algorithm selects the "near post" option, which has a "no shot between posts" 0.78 probability versus a probability of 0.22 in "shot between posts", terminal node. For the "far post" option the probability of "shot between posts" increases to 0.52 while the "no shot between posts" decreases to 0.48.

**Fig 3:**
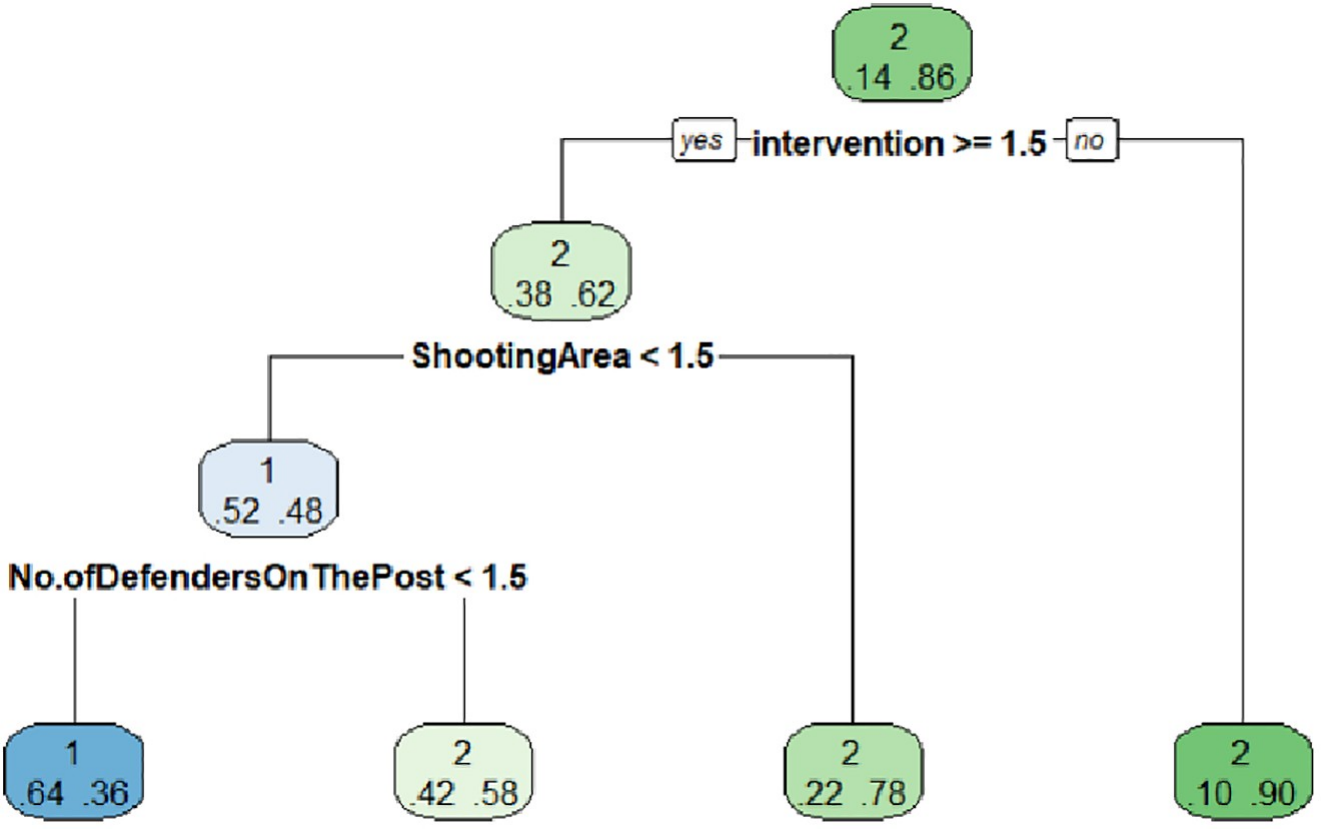
Criterion2 world male shows test.

Last variable included in the tree is "No. of Defenders On The Post", this variable has three categories 1 player (1), 2 players (2) and no players (3), selecting the 1 player (< = 1,5) category, the probability of "shot between posts", is 0.64 and the probability of “no shot between posts” is 0.36. In the case of two or no players, the probability of "shot between posts" is 0.42 and "no shot between posts" is 0.58. Being these last two terminal nodes (Fig 3).

From this tree we are interested in the branch whose terminal node is the realization of the "shot between posts". Starting from a probability of "shot between posts" of 0.14, with an intervention of 3–4 players, combined with a shooting area "far post" and a N°. of Defenders On The Post with 1 player, where the probability of "shot between posts" is increased to 0.64 (Fig 3).

The model predicts 14 "shots between posts" when 14 have actually been observed. Predicts 8 "shots between posts", when they have really been "no shots between posts". As for the "no shot between posts" the model predicts 75 that have actually been "shot between posts", and predicts 527 that they have been "no shot between posts" (Table 4).

**Table 4:** 

Pred. \ Obs.	Shot between posts	No shot between posts
Shot between posts	14	8
No shot between posts	75	527

The effectiveness evaluation of the model, is of 86.70% successes.

### Criterion 2 FIFA Women's World Cup 2015

In female competition, the root node has a probability of "shot between posts" of 0.12 and "no shot between posts" of 0.88. The second node is represented by the variable "intervention" with 3–4 players (> = 1.5). In the event that the "intervention" is not 3–4 players, therefore, is 1–2 players, the probability of "shot between posts" is 0.09, while the "no shot between posts" is 0.91. This node is terminal. And in the case that it is "intervention" with 3–4 players, the probability of "shot between posts" is 0.33 and "no shot between posts" of 0.67.

The third level is formed by the variable "Shooting Area" and the category "near post" as reference, where the probability of "no shot between posts" is 0.55 and that of "shot between posts" of 0.45. In the case of applying the "far post" category, the probability of "no shot between posts" is 0.76, while that of "shot between posts" is 0.24. It is also a terminal node. The next variable included by the algorithm is "Delivery Of The Ball", which has two categories "Direct" (1) and "InDirect" (2), taking as reference the "Direct" category. In the case of "InDirect" the probability of "shot between posts" is 0.31 and "no shot between posts" is 0.69, being a terminal node. For the "Direct" category, the probability of "shot between posts" is 0.54 and "no shot between posts" is 0.46. In this branch of the category "Direct" the variable "Time" is included, which has three categories "0–30" (1), "31–60" (2) and "61–90" (3), and the algorithm takes as criteria the categories "0–30" and "31–60", where the probability of "shot between posts" is 0.65 and that of "no shot between posts" is 0.35.

**Fig 4:**
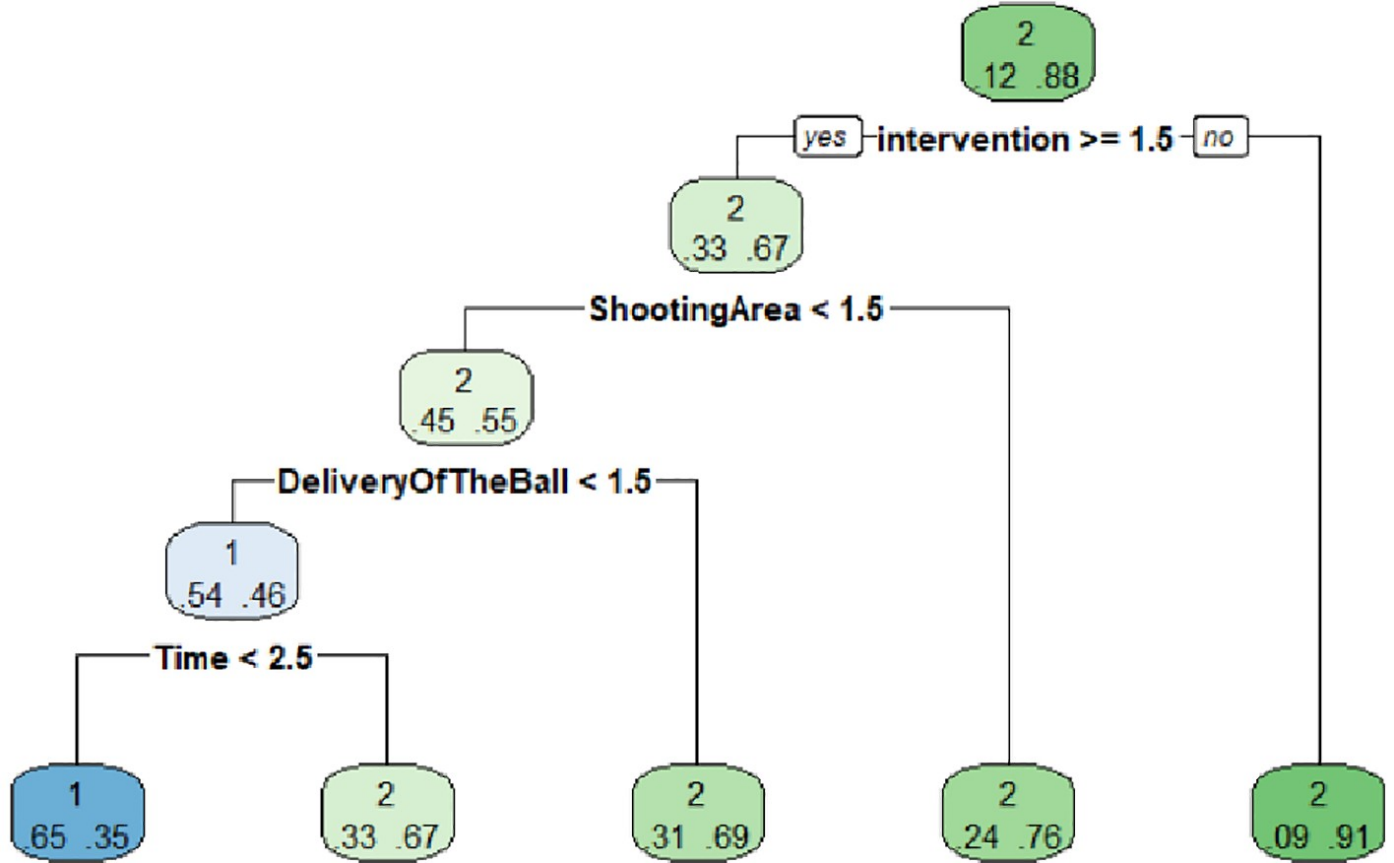
Criterion2 world female shows test.

For the case of the "61–90" category, the probability of "shot between posts" is 0.33, and for "no shot between posts" is 0.67. These last two nodes are terminal (Fig 4).

The most effective model will be one that is executed with the intervention of 3–4 players, the shooting area being near post, delivery of the ball being direct and executed in the first 60 minutes of the match.

The model predicts 1 "shot between posts" when there really is 1 "shot between posts", and predicts 4 "shots between posts" when they really are “no shot between posts”. Predicts "no shot between posts" on 37 occasions when they really are "shots between posts". And predicts 272 "no shot between posts" when they really are not (Table 5).

**Table 5:** 

Pred. \ Obs.	Shot between posts	No shot between posts
Shot between posts palos	1	4
No shot between posts	37	272

The effectiveness evaluation of the model, is of 87.90% successes.

### Criterion 3 FIFA Male World Cup 2014

**Fig 5:**
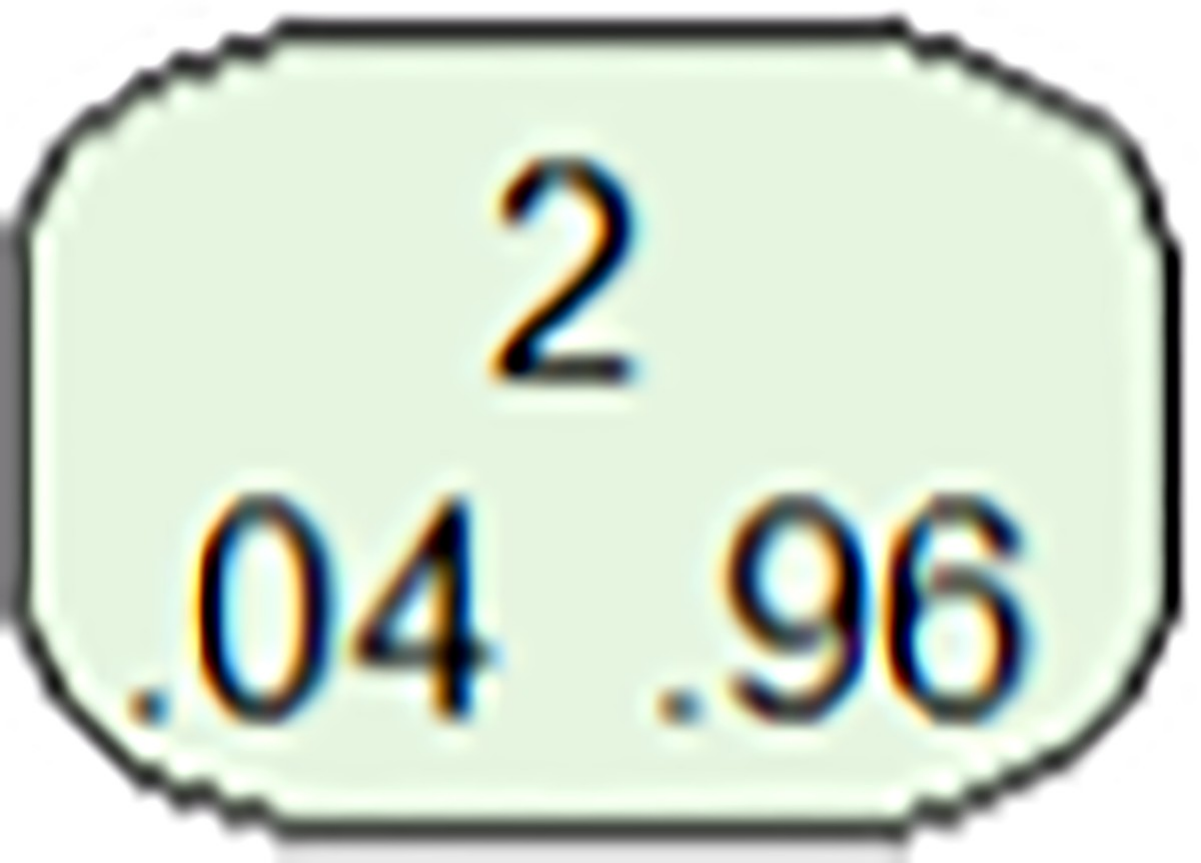
Criterion3 world male shows test.

For criterion 3 "goal" in masculine competition, the basic case of a tree with a single node is obtained. Logically this node is both root and leaf of the tree. The probability of achieving a "goal" is 0.04 and of “no goal”, it is 0.96 (Fig 5).

The model predicts 26 "no goals" when they are really "goals", and 598 "no goals" when they really are not (Table 6).

**Table 6:** 

Pred. \ Obs.	Goal	No Goal
Goal	0	0
No Goal	26	598

The effectiveness evaluation of the model, is of 95.83% successes.

### Criterion 3 FIFA Women's World Cup 2015

**Fig 6:**
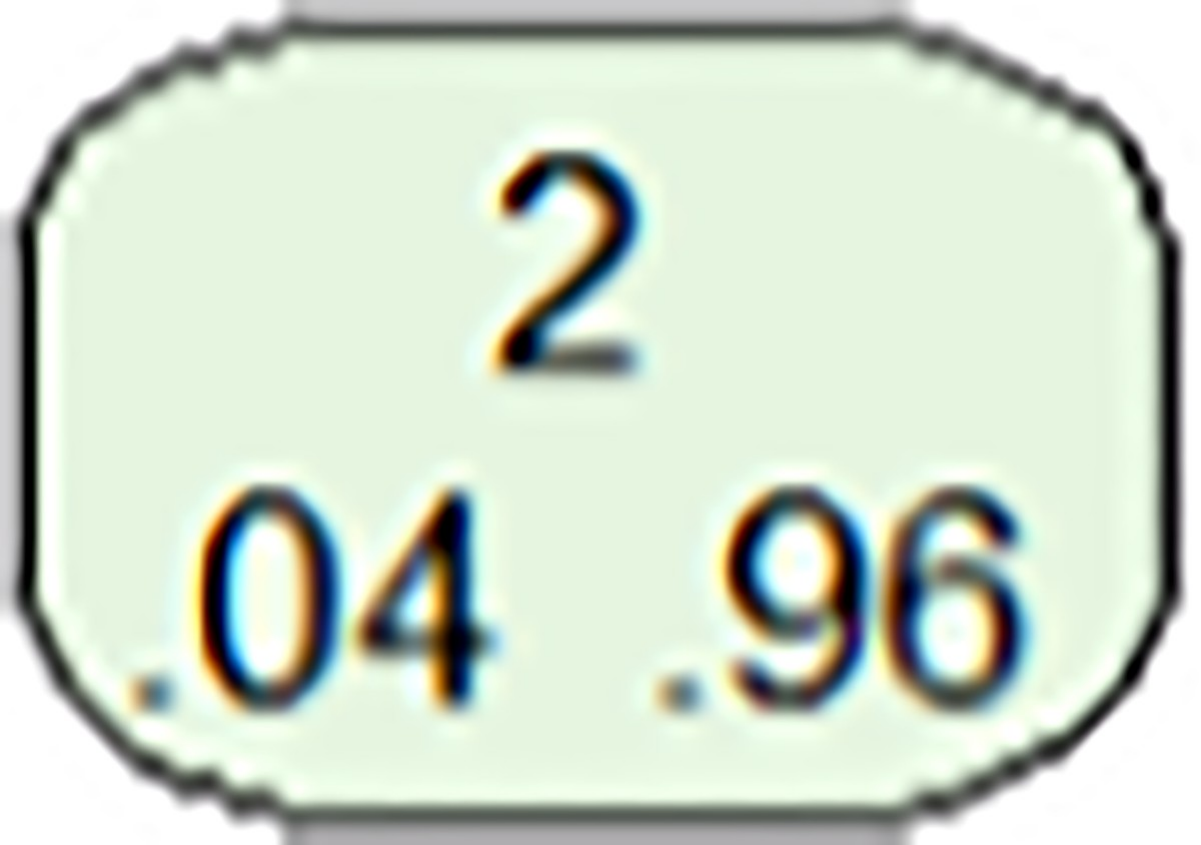
Criterion3 world female shows test.

In women's competition, the probability of getting a "goal" is 0.4 and "no goal" is 0.96 (Fig 6).

The model predicts "no goal" 26 times when it really is "goal" and 695 times "no goal" when it really is "no goal" (Table 7).

**Table 7:** 

Pred. \ Obs.	Goal	No Goal
Goal	0	0
No Goal	26	695

The effectiveness evaluation of the model, is of 96.40% successes.

## Discussion

The main objective of the present study was to know the possible gender differences in one of the more regularly presented static ball actions during matches, such as corner kicks. To do this, the statistical analysis focused on the search of a classification model based on the creation of a decision tree that provides validation tools for exploratory and confirmatory classification analysis, assigning an adequate measurement level to all of the analysis variables. It presents a solution to prediction, classification and segmentation problems, as well as creating a classification model based on flowcharts.

Data analysis started with a decision tree where all variables were treated as nominal, and each node contained a frequency table that showed the number of cases (frequencies and percentages) for each category of the variable explained. The *Chi-squareautomaticinteraction detector* (CHAID) was used as a growth method, consisting of a statistical and multidirectional tree algorithm that explores data quickly and efficiently, and creates segments and profiles with respect to the desired result. In addition, it allows the automatic detection of interactions through Chi-square. In each step, CHAID chooses the predictor variable that presents the strongest interaction with the variable explained. Categories of each predictor merge if they are not significantly different from the predictive variable.

Regarding criterion 1 (shot), the available results show that for both FIFA World Cup variables that behave as efficiency modulators are found. Specifically, the variable that presents the greatest information gain for both championships is "Number of players that intervene on the ball". Specifically, when teams use the association by means of 3–4 players in corner execution, the probability of shot amounts to more than 8 out of 10 corners, which would almost quadruple the probability of shooting in male soccer. On the other hand, as far as women's football is concerned, the intervention of 3–4 players would allow duplicating shooting options in the absence of a model (Fig 2). These data corroborate the works of a multivariate nature described to date [14,20] for men's football, and provide the first data of a multivariate nature for women's football. In addition, corner kicks executed in FWWC2015 presents more variables that modulate the shot and that present information gain. Specifically, when the corner kick is played in the first 30 minutes (0–30 '), and always respecting the intervention of up to 4 players, shot probability would rise to 81%. One of the possible reasons for the high shot probability with these predictor variables may lie in the lack of concentration in the first minutes of the match and the surprise capacity that an execution presents with the intervention of several different players. In this regard, literature shows that in more than 8 out of 10 corners, only 1 or 2 players intervene [12,39], so the interaction between players, proposing a creative attack construction and through association and interaction maneuvers, are fundamental aspects when it comes to reaching a shot.

Finally, the algorithm has also detected information gain with respect to the number of players that defending teams place at the goal posts base to avoid the success of the rival team. This way, when a player who defends the non-observed team is located in this zone with the objective of increasing defensive success, the observed team shot probability is 61%. This data confirms that quality is more important than the number of defenders in achieving defensive success. The main reason for the ineffectiveness of defending with players in these zones is that the most important rule in football, the offside, is eliminated. This way, the attacking team always has a positional and tactical advantage over the defending team, since the option of being in an unregulated position is eliminated.

At the applied level, although in both FIFA World Cup is possible to predict the strength of certain variables as shot predictors, and thus establish tips and tactical alternatives to soccer coaches, the available results allow to think that it is in the FWWC2015 where the algorithm allows to identify more shot modulators and reduce the associated entropy. This will allow to increase and enrich the team’s shot potential when they face these situations.

With regard to the criterion "shot between the three posts", it is possible to verify the corners reduced effectiveness again. The discreet data for both FWC (14% in the case of men's football and 12% in the case of women's football) show the great complexity that this objective presents, since in practically 9 out of 10 corners teams can not shot at goal. It is important to highlight some of the possible evidence that sustain this low efficiency on the part of the executing team, and that show the great entropy that these actions present: execute the corner kick with the precise power and direction; correct timming between the server and the auctioneer; precise organization between the attacking players to avoid losing the ball; approach race, trajectory calculation and shot of the attacker avoiding the obstacles that the defending team may present (type of defense, types of markings, aids …). On the other hand, and as in the shot criterion, the variable that best predicts the shot to goal in both men and women championships is the "number of players that intervene on the ball" (3–4 players). In the case of FWC2014, the variable "shot zone on the 2nd pole" is added as well as the predictive variable "1 defender on the posts", thus increasing the shot probability at 64%. Taking into account the probability of shooting in the absence of a model (14%), based on the information gain the proposed model would quadruple the shooting probabilities among the three posts. These results would allow to corroborate previous works of descriptive [12] and explanatory [20] nature.

As regards FWWC2015, the integrative model capable of explaining and/or predicting the shot between the three posts differs significantly from the male algorithm. In particular, when the corner kick is executed in the first 60 minutes of the game, with the intervention of 3–4 players, the sending is to the first post and is done as a direct sending, the probability of shooting would be five times greater (12% - 65%).

This execution model is the one that presents the lowest degree of entropy among the variables considered, and is the one that best fits the final goal of reaching a shot between the three posts. Although the scarcity of multivariate work in women's football does not allow us to compare the results obtained, if we consider in isolation each variable that has been established as a predictor, it is possible to describe clear differences in comparison to men's soccer. Specifically, as regards the moment of the shot, scientific literature in men's football shows that the last third of the game is suitable [40], as opposed to the first 60 minutes as the model presented evaluated. Regarding the shot area, the scientific debate continues, as there is still no consensus regarding optimal shot zone [12,20,26,41,42].

As for criterion 3 (goal), due to its restrictive nature and high entropy, it is not possible to refer predictive models for either of the male and female championships. The low number of goals that characterizes soccer, where a clear supremacy of the defense over the attack exists (1% of the attacks culminates with obtaining a goal [43], makes it difficult to create predictive models that describe the goal. However, from a researcher perspective, the description of the offensive process and the effectiveness evaluation based on the goals obtained, hardly allows a very limited understanding of its dynamics and team production. Specifically, for the corner kick, criterion 1 (shot) and criterion 2 (shot between the three posts) can be erected as indicators of optimal offensive performance in the success of these actions, because although they are actions that do not cause to obtain a goal immediately, they are capable of inducing a break in the attack/defense balance, creating situations of imminent danger for the opposing team [44].

## Conclusions

The aim of the present study was to propose different success models in corner execution for the FIFA World Cup 2014 and the FIFA Women's World Cup 2015 based on three different criteria: shot, shot between the three posts and goal. The available results have allowed to propose different tactical alternatives for the shot and shot between posts criteria for both championships, and they have also allowed to cement the first results of a multivariate nature in the study of the corner kick in women's soccer. In addition, the present work also highlights the alternative analysis technique based on decision trees, as an alternative to the study of complex dynamic systems such as football. The statistical method based on the creation of decision trees encompasses a set of nonparametric supervised techniques that manage to segment the space of the predictors in simple regions, where it is easier to manage interactions.

## Practical applications and future proposals

Coaches can use these findings to manipulate training tasks related to the management and success of these type of actions, thus increasing the potential for success. On one hand, women team coaches can have new and novel tactical alternatives. On the other hand, know the variables that are conditioning a shot or a goal in corners, will also allow to propose new defensive alternatives to the teams, having information that allows opting for novel defensive mechanisms. Finally, it would be interesting for future research to consider other types of competitions, such as regular championships.

## Supporting information

S1 File Database. (SAV)

## References

[1] Harris S, Reilly T. Space, teamwork and attacking success in soccer In: Reilly T, Lees A, Davis K, Murphy WJ, editors. Science and Football I. (pp. 322–328). London: E and F.N. Spon; 1988. 322–328.

[2] Grant A, Williams M, Reilly T, Borrie T. Analysis of the goals scored in the 1998 World Cup. J Sport Sci. 1999; 17(10): 826–827.

[3] Carling C. Influence of opposition team formation in physical and skill-related performance in a professional soccer team. Eur J Sport Sci. 2011; 11(3): 155–164.

[4] Lago C, Casáis L, Domínguez E, Sampaio J. The effects of situational variables on distance covered at varios speeds in elite soccer. Eur J Sport Sci. 2010; 10(2): 103–109.

[5] Bradley S, Lago-Peñas C, Rey E, Gómez A. The effect of high and low percentage ball possession on physical and technical profiles in English FA Premier League soccer matches. J Sport Sci. 2013; 31(12): 1261–1270. 10.1080/02640414.2013.786185 23697463

[6] Casal C, Maneiro R, Losada J, Ardá T, Marí F. Possession zone as a performance indicator in football. The game of the best teams. Front Psychol. 2017; 8: 1176 10.3389/fpsyg.2017.01176 28769833 PMC5509942

[7] Di Salvo V, Baron R, Tschan H, Montero C, Bachl N, Pigozzi F. Performance characteristics according to playing position in elite soccer. Int J Sports Med. 2007; 28(03): 222–227. 17024626 10.1055/s-2006-924294

[8] Castañer M, Barreira D, Camerino O, Anguera T, Fernandes T, Hileno R. Mastery in goal scoring, T-pattern detection and polar coordinate analysis of motor skills used by Lionel Messi and Cristiano Ronaldo. Front Psychol. 2017; 8:741 10.3389/fpsyg.2017.00741 28553245 PMC5427849

[9] Duch J, Waitzman S, Amaral L. Quantifying the Performance of Individual Players in a Team Activity. PloS ONE. 2010; 5(6): 1–7. 10.1371/journal.pone.0010937 PMC2886831 20585387

[10] McGarry T, Anderson D, Wallace S, Hughes M, Franks I. Sport competition as a dynamical self-organizing system. J Sports Sci. 2002; 20(10): 771–781. 10.1080/026404102320675620 12363294

[11] Yiannakos A, Armatas V. Evaluation of the goal scoring patterns in European Championship in Portugal 2004. Int J Perform Anal Sport. 2006; 6(1): 178–188.

[12] Carling C, Williams M, Reilly T. Handbook of soccer match analysis: A systematic approach to improving performance. Abingdon, UK: Routledge; 2005.

[13] Wallace J, Norton K. Evolution of World Cup soccer final games 1966–2010: Game structure, speed and play patterns. J Sci Med Sport. 2014; 17(2): 223–228. 10.1016/j.jsams.2013.03.016 23643671

[14] Casal C, Maneiro R, Ardá T, Losada J, Rial A. Analysis of Corner Kick Success in Elite Football. Int J Perform Anal Sport. 2015; 15: 430–451.

[15] Sainz de Baranda P, López-Riquelme D. Analysis of corner kicks in relation to match status in the 2006 World Cup. Eur J Sport Sci. 2012; 12(2): 121–129.

[16] Kjær J, Agergaard S. Understanding women’s professional soccer: the case of Denmark and Sweden. Soccer Society. 2013; 14(6): 816–833.

[17] Mara J, Wheeler K, Lyons K. Attacking Strategies That Lead to Goal Scoring Opportunities in High Level Women´s Football. In J Sports Sci Coach. 2012; 7(3): 565–577.

[18] Östenberg A, Roos H. Injury risk factors in female European football. A prospective study of 123 players during one season. Scand J Med Sci Sports. 2000; 10(5): 279–285. 11001395 10.1034/j.1600-0838.2000.010005279.x

[19] Maneiro R. (2014). Analysis of set plays in high performance soccer: corner kick and indirect free kick. An attempt to identify explanatory variables. Thesis, University of A Coruña. 2014. Available from: http://ruc.udc.es/dspace/handle/2183/12426

[20] Ardá T, Maneiro R, Rial A, Losada J, Casal C. Análisis de la eficacia de los saques de esquina en la copa del mundo de fútbol 2010. Un intento de identificación de variables explicativas. J Sport Psychol. 2014; 23(1): 165–172.

[21] Pan S, Huang H, Ding J, Zhang W, Tomlin C. Pursuit, evasion and defense in the plane. In American Control Conference (ACC). 2012: 4167–4173.

[22] Acar MF, Yapicioglu B, Arikan N, Yalcin S, Ates N, Ergun M. Analysis of goals scored in the 2006 world cup In T Reilly, Korkusuz F, editors. Science and football VI. London: Routledge; 2009 pp. 233–242.

[23] Siegle M, Lames M. Games interruptions in elite soccer. J Sports Sci. 2012; 30(7): 619–624. 10.1080/02640414.2012.667877 22404095

[24] Pulling C, Robins M, Rixon T. Defending Corner Kicks: Analysis from the English Premier League. Int J Perform Anal Sport. 2013;13(1): 135–148.

[25] Schmicker H. An Application of SaTScan to Evaluate the Spatial Distribution of Corner Kick Goals in Major League Soccer. Int JComput Sci Sport. 2013;12(2): 70–79.

[26] Maneiro R, Ardá T, Rial A, Losada JL, Casal CA, López-García S. Análisis descriptivo y comparativo de los saques de esquina. UEFA Euro 2012. Rev And Med Dep. 2017; 10(3): 95–99.

[27] Casal CA, Losada JL, Maneiro R, Ardá T. Influencia táctica del resultado parcial en los saques de esquina en fútbol / Influence of Match Status on Corner Kick in Elite Soccer. Rev Int Med Cien Act Física Dep. 2017; 17(68): 715–728.

[28] Silva P, Vilar L, Davids K, Araújo D, Garganta J. Sports teams as complex adaptive systems: manipulating player numbers shapes behaviours during football small-sided games. SpringerPlus. 2016; 5(1): 191. 27026887 10.1186/s40064-016-1813-5 PMC4769238

[29] Balagué N, Torrents C, Hristovski R, Kelso J. (2017). Sport science integration: An evolutionary synthesis. Eur J Sport Sci. 2017; 17(1): 51–62. 10.1080/17461391.2016.1198422 27685425

[30] Anguera MT. Observational Typology. Qual Quant. 1979; 13(6): 449–484.

[31] Anguera MT, Blanco-Villaseñor A, Losada JL. Observational designs, key issue in the process of observational methodology. Methods Behav Sci. 2001; 3(2): 135–161.

[32] Sánchez-Algarra P, Anguera MT. Qualitative/quantitative integration in the inductive observational study of interactive behaviour: Impact of recording and coding predominating perspectives. Qual Quant. 2013; 47(2): 1237–1257.

[33] Manolov R, Losada JL. Simulation theory applied to direct systematic observation. Front Psycho. 2017;8: 905 10.3389/fpsyg.2017.00905 PMC5462976 28642721

[34] Losada JL, Manolov R. The process of basic training, applied training, maintaining the performance of an observer. Qual Quant. 2015; 49(1): 339–347.

[35] Gabin B, Camerino O, Anguera MT, Castañer M. Lince: Multiplatform sport analysis software. Procedia Soc Behav Sci. 2012; 46: 4692–4694.

[36] Fleiss JL, Levin B, Paik MC. Statistical methods for rates and proportions. 3rd ed Hoboken: John Wiley y Sons; 2003.

[37] Shannon CE. A Mathematical Theory of Communication. Bell System Technical Journal. 1948; 27 (4), 623–65.

[38] Quinlan JR. Programs for Machine Learning. Morgan Kaufmann: San Francisco; 1993

[39] Pulling C. Long corner kicks in the English Premier League: Deliveries into the goal area and critical area. Kinesiol. 2015; 47(2): 193–201.

[40] Armatas V, Yiannakos A, Sileloglou P. Relationship between time and goal scoring in soccer games: Analysis of three Worlds Cups. Int J Perform Anal Sport. 2007; 7(2), 48–58.

[41] Page R, Robins M. A corner kick analysis of a League One professional football team. Int J Perform Anal Sport. 2012; 12(3), 793.

[42] Maneiro R. y Amatria M. Polar coordinate analysis of relationships with teammates, areas of the pitch, and dynamic play in soccer: a study of Xabi Alonso. Front. Psychol. 2018; 9:389 10.3389/fpsyg.2018.00389 29628905 PMC5876316

[43] Garganta J. Modelaçao táctica do jogo de futebol. Estudo da Organizaçao da Fase Ofensiva em Equipas de Alto Rendimento [The Study of the Organization of the Offensive Phase in Elite Soccer Teams]. Thesis, University of Porto. 1997.

[44] Peñas C, Fraga F, Graña M, Codesido J. Evaluación de las acciones ofensivas en el fútbol de rendimiento mediante indicadores de éxito en diseños diacrónicos intensivos retrospectivos. Apunts. 2003; 2(72): 96–102.

